# Examining mortality among formerly homeless adults enrolled in Housing First: An observational study

**DOI:** 10.1186/s12889-015-2552-1

**Published:** 2015-12-04

**Authors:** Benjamin F. Henwood, Thomas Byrne, Brynn Scriber

**Affiliations:** School of Social Work, University of Southern California, 1150 S. Olive Street, 14th Floor, Los Angeles, CA 90015-2211 USA; School of Social Work, Boston University, 264 Bay State Rd., Boston, MA 02215 USA; Pathways to Housing PA, 5201 Old York Rd., 4th Floor, Philadelphia, PA 19141 USA

**Keywords:** Homelessness, Housing First, Health disparities, Vulnerability index, Death, Permanent supportive housing

## Abstract

**Background:**

Adults who experience prolonged homelessness have mortality rates 3 to 4 times that of the general population. Housing First (HF) is an evidence-based practice that effectively ends chronic homelessness, yet there has been virtually no research on premature mortality among HF enrollees. In the United States, this gap in the literature exists despite research that has suggested chronically homeless adults constitute an aging cohort, with nearly half aged 50 years old or older.

**Methods:**

This observational study examined mortality among formerly homeless adults in an HF program. We examined death rates and causes of death among HF participants and assessed the timing and predictors of death among HF participants following entry into housing. We also compared mortality rates between HF participants and (a) members of the general population and (b) individuals experiencing homelessness. We supplemented these analyses with a comparison of the causes of death and characteristics of decedents in the HF program with a sample of adults identified as homeless in the same city at the time of death through a formal review process.

**Results:**

The majority of decedents in both groups were between the ages of 45 and 64 at their time of death; the average age at death for HF participants was 57, compared to 53 for individuals in the homeless sample. Among those in the HF group, 72 % died from natural causes, compared to 49 % from the homeless group. This included 21 % of HF participants and 7 % from the homeless group who died from cancer. Among homeless adults, 40 % died from an accident, which was significantly more than the 14 % of HF participants who died from an accident. HIV or other infectious diseases contributed to 13 % of homeless deaths compared to only 2 % of HF participants. Hypothermia contributed to 6 % of homeless deaths, which was not a cause of death for HF participants.

**Conclusions:**

Results suggest HF participants face excess mortality in comparison to members of the general population and that mortality rates among HF participants are higher than among those reported among members of the general homeless population in prior studies. However, findings also suggest that causes of death may differ between HF participants and their homeless counterparts. Specifically, chronic diseases appear to be more prominent causes of death among HF participants, indicating the potential need for integrating medical support and end-of-life care in HF.

**Electronic supplementary material:**

The online version of this article (doi:10.1186/s12889-015-2552-1) contains supplementary material, which is available to authorized users.

## Background

Adults who experience prolonged homelessness have mortality rates 3 to 4 times that of the general population [1–3], and communities including New York City [4] and Philadelphia [5] have enacted surveillance systems to monitor and address mortality in this population. Injuries, substance abuse, heart disease, liver disease, and ill-defined conditions have been reported as accounting for the vast majority of deaths among individuals experiencing homelessness [1, 3]. Housing can protect against exposure to weather, infections, drugs, and violence experienced while living on the streets. There is some evidence that exiting homelessness to housing is associated with reduced risk of mortality [6], but whether access to housing affects health disparities, including mortality rates of individuals who have experienced long-term homelessness in particular, is unclear [7].

Housing First (HF) is an evidence-based practice that addresses homelessness by offering immediate access to housing while providing ongoing community-based support services [8]. HF has been adopted in multiple countries including the United States [9], Canada [10], Europe [11], and Australia [12], and effectively ends homelessness for people who have experienced a lifetime of cumulative adversity [13] and carry a significant disease burden based on multiple risk categories [14]. To date, however, there has been no research on premature mortality among formerly homeless adults who have enrolled in HF. In the United States, this gap in the literature exists despite research that suggests chronically homeless adults constitute an aging cohort; nearly half are aged 50 years old or older [15].

To begin to address this gap, the present study explored mortality among formerly homeless adults who moved into housing as part of an HF program in Philadelphia, PA. We examined death rates and causes of death among HF participants. We then compared HF participant mortality to two groups: members of the general population and the homeless population. We also compared the causes of death and characteristics of decedents in the HF program to a sample of adults identified as homeless at the time of death through formal review process in Philadelphia.

## Methods

We used administrative records from the HF program to identify a cohort of 292 formerly homeless individuals who moved into a housing unit between September 2008, when the HF program first began operations, and October 2013. Individuals who had been admitted to the HF program but had not yet moved into housing were excluded from the study cohort, because these individuals could still be considered homeless. In 2014, HF medical and continuous quality improvement staff members reviewed and documented the events that preceded the death of all participants who died during the first 6 years of the program’s operation (2008–2013) for purposes of program improvement. These data were used to ascertain the date and cause of death among HF participants. Members of the study cohort were followed prospectively from the initial date of their move to a housing unit until either their date of death or October 31, 2013; this observation period was measured in person-years.

We conducted analyses to examine mortality among HF participants from several perspectives. First, we calculated all-cause and cause-specific mortality rates, expressed as deaths per 100,000 person-years of observation, for the entire study cohort. Second, we used survival analysis methods to assess the risk and predictors of death following HF participants’ move to housing. We estimated hazard functions and Kaplan-Meier survival curves to conduct descriptive analyses of the timing and occurrence of death following move to housing and fitted a Cox proportional hazards regression model to assess the relationship between HF participants’ demographic characteristics (gender, race and age) and risk of death following move to housing.

Third, we calculated all-cause mortality rates among HF participants stratified by age and sex. We did not further stratify these age- and gender-specific mortality rates by cause due to sparse data. We used mortality rate ratios to compare the age- and sex-specific all-cause mortality rates among HF participants to members of the general population in Philadelphia between 2008 and 2013. To calculate these rate ratios, we divided the all-cause mortality rate among members of the study cohort by the corresponding rates in the general population. These values were adjusted for race using direct standardization, with the Philadelphia general population serving as the standard population. We calculated 95 % confidence intervals for these rate ratios using established methods [16]. We obtained mortality data for the Philadelphia general population (2008–2013) from the CDC Wide-ranging Online Data for Epidemiologic Research compressed mortality files regarding underlying cause of death [17].

Fourth, we compared mortality rates in our sample of HF participants to mortality rates of individuals experiencing homelessness as reported in prior studies. To achieve this, we identified published studies that provided mortality rates or information from which such rates could be calculated. We only included studies that were conducted in North America. We identified 10 studies [3, 6, 18–25] that met these criteria. We excluded three studies: one study [24] because it only reported data on homeless youths younger than 25; a second [18] because it grouped individuals living in emergency shelters with those living in rooming houses and hotels; and a third [25] because it only reported information for individuals experiencing homelessness as part of a family with children. Following a previously employed approach for comparing mortality rates among homeless individuals across several studies [20, 23], we obtained or calculated age-specific all-cause mortality rates for each identified study using age groupings that were as similar as possible (younger, middle-aged, older). We then calculated mortality rate ratios by comparing the age-specific all-cause mortality rates observed among HF participants in the present study with those obtained or calculated from the identified studies. We calculated 95 % confidence intervals for these rate ratios when possible using published data. These rates and rate ratios were not adjusted for race.

Finally, we compared the causes of death and characteristics of decedents in the HF program with information on individuals identified as homeless at their time of death in Philadelphia using data from a report by the City of Philadelphia’s Homeless Death Review Team [5]. Homeless status in the report is determined using the U.S. Department of Housing and Urban Development’s definition of homelessness, which considers individuals to be homeless if they are residing in an emergency shelter or in a place not meant for human habitation (i.e., unsheltered or “street” homelessness). Although the report included sheltered and unsheltered decedents, it did not provide specific information about the living situation of decedents at the time of their death. The report, which identified 90 individuals who died while homeless during a 2-year period (2009 and 2010) that overlaps with the follow-up period for the HF participant cohort, provided demographic characteristics from the medical examiner’s office that included age, gender, and race. The medical examiner also classified the manner of death as homicide, suicide, accidental, natural, or undetermined. A natural manner of death includes infectious diseases, cardiovascular or other chronic conditions, and cancers. The specific primary cause of death was also noted and included: specific disease (e.g., infectious, circulatory, respiratory), drug intoxication or alcoholism, injury (e.g., blunt force, gunshot wound), cancer, hyper- or hypothermia, HIV, or other. To facilitate comparisons, the demographic information and manner and cause of death among HF decedents were reclassified using categories reported in the City of Philadelphia’s report. The report did not include information about the size of the overall homeless population in Philadelphia during 2009 and 2010, nor are we aware of another publicly available source that provides such information. As such, it was not possible to calculate mortality rates for the Philadelphia homeless population using data from the report; consequently, comparisons between the HF and homeless group were conducted using chi-square and Fisher’s exact tests. The small number of deaths that occurred among HF participants during the same time frame as the City of Philadelphia’s report (i.e., 2009 and 2010) precluded a comparison of deaths between the same groups during the same time period. Instead, we opted to compare HF deaths observed during the entire study period (i.e., 2008–2013) with those identified in the report. Study protocols were found to be exempt by the Pathways to Housing, Inc.’s institutional review board.

## Results

Table 1 presents the characteristics of the 292 individuals in the overall HF participant cohort and decedents. The mean age at move to housing was 51.3, and roughly 80 % of the study cohort was between the ages of 45 and 74 at move to housing. The study cohort was predominantly male (70 %) and African American (68 %). The median duration of follow-up was 3.2 years, resulting in 1045 person-years of observation. Forty-one deaths occurred during the study period, with a mean age at death of 57.2 years. The majority of decedents were male (78 %) and African American (59 %).

**Table 1:** Characteristics of all Housing First participants in study cohort (*N* = 292) and decedents (*N* = 41)

	Overall	Decedents
	*n* (%)	*n* (%)
Gender		
Female	84 (28.8)	9 (22.0)
Male	207 (70.9)	32 (78.0)
Unknown	1 (0.3)	0 (0.0)
Age^a^		
19–44	58 (19.9)	4 (9.8)
45–64	213 (72.9)	32 (78.0)
65–74	21 (7.2)	5 (12.2)
Race		
Black	197 (67.5)	24 (58.5)
White	78 (26.7)	17 (41.5)
Other	17 (5.8)	0 (0.0)

^a^Figures in this row reflect *M* (range) at time of move to housing for the overall sample and at time of death for decedents

As shown in Table 2, the crude mortality rate for the study cohort was 3916.1 deaths per 100,000 person-years. Disease of the circulatory system was the leading cause of death, accounting for 29.3 % of deaths in the study cohort. Cancer accounted for 22 % of deaths, whereas drugs or alcohol caused approximately 10 % of deaths. Kidney and respiratory disease caused about 5 % of deaths each, with diabetes, HIV, injury, and liver disease each accounting for about 2 % of deaths.

**Table 2:** Cause of death among Housing First decedents and crude mortality rates

Cause of death	Number of deaths	% of deaths	Crude mortality rate per 100,000 person years
All causes	41	100.0	3916.1
Circulatory system disease	12	29.3	1146.2
Cancer	9	22.0	859.6
Other	8	19.5	764.1
Drugs or alcohol	4	9.8	382.1
Kidney disease	2	4.9	191.0
Respiratory disease	2	4.9	191.0
Diabetes	1	2.4	95.5
HIV	1	2.4	95.5
Injury	1	2.4	95.5
Liver disease	1	2.4	95.5

**Fig. 1:**
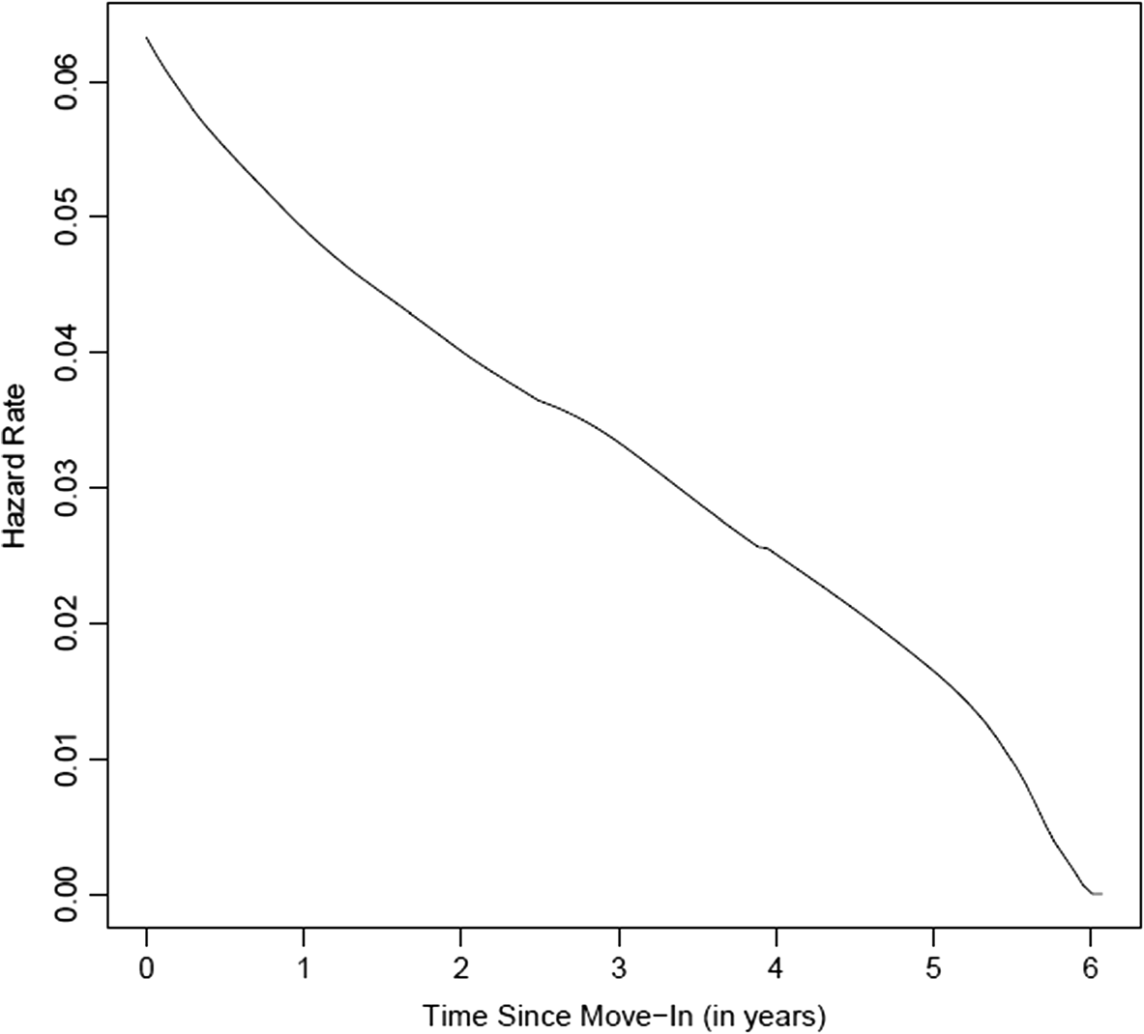
Hazard function for death following move to housing among Housing First participants

Figure 1 presents the estimated hazard function for death following HF participants’ move to housing. The hazard for death was highest in the period directly following participants’ move to housing and then declined steeply and steadily thereafter. Among decedents, the median time to death following move to housing was 1.3 years, and 25 % of deaths occurred within the first 6 months following entry into housing. Kaplan-Meier 1-, 3-, and 5-year survival rates among all members of the HF participant cohort were 94.5 % (95 % CI 91.9–97.2 %), 88.3 % (95 % CI 84.6–92.3 %), and 82.9 % (95 % CI 77.9–88.2 %), respectively. Only age was a significant predictor in the Cox regression model, with those in the 65–74 age bracket having almost a five-fold increase (HR 4.8, 95 % CI 1.2–18.1) in the risk of death following their initial move to housing.

Table 3 presents age, gender, and overall all-cause mortality rates and rate ratios (RRs) comparing mortality rates in the HF participant cohort with those of the general population of Philadelphia. The all-cause mortality rate among male HF participants in the 45–64 age bracket was 4.7 times higher than in the general population (RR 4.7, 95 % CI 2.1–10.8). Estimates of the risk ratios for all other age and gender subgroups exceeded 1, but none of these differences was statistically significant. However, the all-cause mortality rates were higher for male HF participants (RR 4.4, 95 % CI 1.7–11.7) and all HF participants (RR 4.6, 95 % CI 1.6–13.2) relative to the Philadelphia general population.

**Table 3:** Mortality rates and rate ratios comparing Housing First participants and the general population in Philadelphia

	Deaths	Person-Years of Observation	CR^a^	Race-Adjusted RR^b^	95 % CI
Men					
25–44	2	114	1754.4	8.1	0.2, 334.7
45–64	26	554	4693.1	4.7	2.1, 10.8
65–74	4	56	7142.9	2.3	0.6, 9.2
All men^c^	32	725	4413.8	4.4	1.7, 11.7
Women					
25–44	1	76	1315.8	23.1	0, 10,988.9
45–64	5	195	2564.1	2.8	0.7, 11.2
65–74	3	49	6122.4	2.1	0.5, 9.8
All women^c^	9	320	2812.5	4.8	0.6, 39.1
Total^c^	41	1045	3923.4	4.6	1.6, 13.2

Abbreviations: CR, crude rate; CI, confidence interval; RR, rate ratio

^a^Deaths per 100,000 person-years of observation

^b^Mortality rate ratios calculated by dividing the race-adjusted mortality rates for the Housing First participant cohort by corresponding mortality rates in the Philadelphia general population. Race-adjusted mortality rates were calculated using direct standardization with the Philadelphia general population during the study period (2003–2013) used as the standard population

^c^Mortality rate ratios also adjusted for age using direct standardization with the Philadelphia general population during the study period used as the standard population

Additional file 1 presents the results of comparisons of mortality rates observed among HF participants in the current study and the corresponding mortality rates for members of the homeless population in several North American cities reported in previously published studies. Point estimates of the mortality risk ratios show that mortality rates among HF participants in the present study were generally higher than those documented in prior studies for homeless individuals in similar age brackets. For most age and gender subgroups, these risk ratios suggest that mortality rates among HF participants in the present study were between 1.2 and 3 times higher than those among their homeless counterparts. However, in cases in which it was possible to conduct tests of statistical significance, the only significant difference in mortality rates was found in a comparison of middle-aged male HF participants, who had a increased risk of mortality (RR 2.2, 95 % CI 1.5–3.2) relative to homeless men in the same age bracket from a study using data from New York City [6].

Table 4 presents the comparison between the 41 HF participants who died during the first 6 years of the program’s operation and the homeless decedents identified by the City of Philadelphia’s Homeless Death Review Team during an overlapping 2-year time period. The majority of decedents in both the HF and homeless groups were between the ages of 45 and 64 at their time of death, although there were proportionally more decedents younger than 45 in the homeless group. Among those in the HF group, 78 % died from natural causes, compared to 49 % in the homeless group. This included 22 % of HF participants as opposed to 7 % in the homeless group who died from cancer. Among homeless adults, 40 % died from an accident, which was significantly more than the 12 % of HF participants who died from an accident. An infectious disease other than HIV caused more than 1 in 10 homeless deaths and hypothermia caused an additional 6 % of deaths; neither of these factors contributed to the death of HF participants.

**Table 4:** Comparison between decedents in a Housing First program in Philadelphia (2008–2013) and individuals identified as homeless at time of death in Philadelphia (2009–2010)

	Housing First	Homeless	
	*n* (%)	*n* (%)	*p*
Gender			.630
Male	32 (78.0)	75 (83.3)	
Female	9 (22.0)	15 (16.7)	
Age			.088
< 25	0 (0.0)	3 (3.3)	
25–34	1 (2.4)	5 (5.6)	
35–44	2 (4.9)	9 (10.0)	
45–54	10 (24.4)	34 (37.8)	
55–64	21 (51.2)	22 (24.4)	
65–74	7 (17.1)	14 (15.6)	
75+	0 (0.0)	3 (3.3)	
Manner of death			< .001
Accident	5 (12.2)	36 (40.0)	
Homicide	1 (2.4)	8 (8.9)	
Suicide	0 (0.0)	2 (2.2)	
Natural	32 (78.0)	44 (48.9)	
Other or unknown	3 (7.3)	0 (0.0)	
Cause of death			< .001
Drug or alcohol	4 (9.8)	23 (25.6)	
Circulatory system disease	12 (29.3)	21 (23.3)	
Injury	1 (2.4)	13 (14.4)	
HIV and infectious disease	1 (2.4)	12 (13.3)	
Cancer	9 (22.0)	6 (6.7)	
Hypothermia	0 (0.0)	5 (5.6)	
Respiratory disease	2 (4.9)	3 (3.3)	
Fire	0 (0.0)	3 (3.3)	
Diabetes	1 (2.4)	0 (0.0)	
Other	11 (26.8)	4 (4.4)	

## Discussion

This study is the first to our knowledge to examine mortality among formerly homeless participants in an HF program. Overall, the results from this study are consistent with prior research on early mortality among populations that have experienced long-term homelessness [1, 20, 22] and suggest that adverse health outcomes associated with homelessness persist even after individuals obtain housing. Importantly, we found that risk of death among HF participants residing in housing was highest during the period immediately following their initial entry into housing. On one hand, this may reflect particularly heightened vulnerability and poor health in a certain segment of individuals who die shortly after entering housing. On the other hand, this finding may indicate that the period of transition into housing is one of elevated risk, during which it is of great importance to help individuals access needed health care and other services that may help prevent potentially avoidable deaths.

Comparisons of mortality rates among members of the HF study cohort with previously reported mortality rates in the homeless population in several North American cities also provide some evidence that formerly homeless HF participants have excess mortality in comparison to the more general homeless population. This finding is not entirely unexpected because individuals experiencing chronic homelessness, who have been shown to have more complex health and behavioral health problems than their homeless peers who are not chronically homeless [26], are the target population for HF programs. Put differently, HF program participants are typically members of the homeless population who have the highest risk of mortality. Future studies should contrast the mortality rates of HF participants with members of the homeless population who experience chronic homelessness. This would provide a better sense of the impact of HF on housing mortality, but such a comparison was not possible with available data. Thus, a more rigorous assessment of the impact of HF on mortality is an important goal for future research.

Findings from this study with respect to the causes of death among HF participants are also noteworthy. Circulatory system disease was the leading cause of death among members of the HF study cohort, accounting for almost 30 % of deaths, followed by cancer, which accounted for 22 % of deaths in the study cohort. These two causes combined with kidney disease, respiratory disease, diabetes, HIV, and liver disease to account for 78 % of deaths in the HF study cohort. In contrast, drug- and alcohol-related causes and injury accounted for only 12 % of deaths. As a point of comparison, a recent study found drug overdose to be the leading cause of death among homeless adults in Boston [21], accounting for 17 % of deaths, with cancer and heart disease each accounting for about 16 % of deaths. Furthermore, the comparison of HF decedents with those identified by the Philadelphia Homeless Death Review Team shows that drug, alcohol, injury, and accident were more prominent causes of death in the latter group. Similarly, comparisons of the manner of death indicate that a much greater proportion of deaths among homeless decedents in Philadelphia were due to accident or homicide relative to members in the HF cohort. Taken together, these findings suggest that HF participants and their currently homeless counterparts may face different mortality-related risks.

Elevated rates of accidental deaths, homicide, and deaths from infectious diseases in the homeless group may reflect the fact that homelessness increases exposure to risks and unmet service needs, which supports the notion that HF may serve as a protective factor against some causes of death. Nonetheless, HF participants were more likely to die of natural causes, potentially reflecting underlying differences in the disease burden of these two groups, which could be explained by a growing practice in the United States known as *vulnerability indexing* wherein homeless individuals identified as having medical conditions placing them at the highest risk of death receive priority for placement in permanent housing programs [27]. This practice, which was implemented in Philadelphia starting in 2011, suggests that HF participants are more vulnerable to death than those who remain on the streets, in which case any evidence supporting the notion that HF serves as a protective factor is understated.

The high number of deaths in the HF group resulting from chronic diseases also suggests that HF providers may need to reorient their supportive service delivery models, which have traditionally focused on housing stability and behavioral health interventions, to increasingly focus on chronic disease management and end-of-life care [28, 29]. This may entail additional staff training on integrated care models [30, 31] to address client needs. Growing interest in the use of newly available Medicaid funds via the Affordable Care Act to offer supportive services in permanent supportive housing programs could present an important opportunity for HF programs to develop new service models [32]. It may also be important to provide increased support to help staff members handle the emotional impact of client deaths at a time when HF may have provided renewed hopes of recovery from chronic homelessness. Interventions designed for health care professionals who encounter patient deaths may be useful models [33].

This is the first study to consider premature mortality among formerly homeless adults who have enrolled in Housing First, an approach that has been adopted as the official policy of the United States to address chronic homelessness [9] and is being implemented in multiple countries [10–12]. The use of death reviews conducted by medical professionals for both homeless adults and HF participants in the same city during the same time period is a strength of the study. The small sample size of the HF participant cohort represents a limitation of the study, particularly regarding comparisons of mortality rates among HF participants with those among members of the general population. Lack of more detailed information about the health conditions of HF participants at enrollment and other characteristics that may be related to mortality risk is also a serious limitation in the present study. Interpretation of the results of the comparison between HF decedents and those identified in Philadelphia Homeless Death Review study warrants caution for several reasons. First, because only three deaths occurred among HF participants during the time period covered in the Philadelphia Homeless Death Review report, it was necessary to compare HF decedents identified during a 6-year period with homeless decedents identified during a 2-year period. Moreover, because data on the size and characteristics of the overall Philadelphia homeless population during the time period were not covered by the report, it was not possible to calculate mortality rates in the homeless population during this time period and compare them to those observed among HF participants. Finally, the absence of information about whether homeless decedents identified in the death review report were eligible for or offered HF services represents a clear limitation.

## Conclusions

HF may decrease mortality rates for adults who have experienced chronic homelessness by reducing exposure to risks while homeless that contribute to higher rates of deaths caused by accidents, homicide, and infectious diseases. This idea is further supported when considering that individuals who are most medically vulnerable are often prioritized for HF, which may also account for higher rates of HF participant deaths due to natural causes. Integrating medical support and end-of-life care in HF support services is needed, as is support for staff members who are working to promote recovery among highly vulnerable individuals.

## Additional files

Additional file 1:
**Mortality rates and rate ratios comparing Housing First participants in study cohort and individuals experiencing homelessness.** (DOCX 25 kb)
