# Supplementary material for: Examining mortality among formerly homeless adults enrolled in Housing First: An observational study
Source: BMC Public Health. 2015 Dec 4;15:1209. doi: 10.1186/s12889-015-2552-1 (PMC4669629; doi:10.1186/s12889-015-2552-1)
Supplement: Additional file 1: — Mortality rates and rate ratios comparing Housing First participants in study cohort and individuals experiencing homelessness. (DOCX 25 kb) [file 12889_2015_2552_MOESM1_ESM.docx]

**Additional file 1 – Mortality rates and rate ratios comparing Housing First participants in study cohort and individuals experiencing homelessness**

|  |  | Young | | | | Middle-aged | | | | Older | | | |
| --- | --- | --- | --- | --- | --- | --- | --- | --- | --- | --- | --- | --- | --- |
| City and study | Period | Age | CR^a^ | RR^b^ | 95% CI | Age | CR^a^ | RR^b^ | 95% CI | Age | CR^a^ | RR^b^ | 95% CI |
| *Men* |  |  |  |  |  |  |  |  |  |  |  |  |  |
| Philadelphia (current) | 2008–2013 | 25–44 | 1,754 | -- | -- | 45–64 | 4,693 | -- | -- | 65–74 | 7,143 | -- | -- |
| Boston [19] | 1988–1993 | 25–44 | 1,218 | 1.4 | -- | 45–64 | 2,170 | 2.2 | -- | -- | -- | -- | -- |
| Boston [21] | 2003–2008 | 25–44 | 950 | 1.8 | -- | 45–64 | 23,378 | 2 | -- | 65–84 | 4,051 | 1.8 | -- |
| New York [22] | 1987–1994 | 25–44 | 1,220 | 1.4 | 0.4, 5.8 | 45–64 | 3,255 | 1.4 | 0.9, 2.3 | 65–74 | 5,738 | 1.2 | 0.4, 4.1 |
| New York [23] | 1990–2008 | 25–44 | 902 | 1.9 | 0.5, 7.7 | 45–64 | 2,170 | 2.2 | 1.5, 3.2 | 65–74 | 5,314 | 1.3 | 0.5, 3.5 |
| Philadelphia [3] | 1985–1988 | 35–54 | 1,290 | 1.4 | 0.3, 5.9 | -- | -- | -- | -- | 55–74 | 2,703 | 2.6 | 0.7, 9.5 |
| Toronto [20] | 1995–1977 | 25–44 | 669 | 2.6 | -- | 45–64 | 1,680 | 2.8 | -- | -- | -- | -- | -- |
| *Women* |  |  |  |  |  |  |  |  |  |  |  |  |  |
| Philadelphia (current) | 2008–2013 | 25–44 | 1,316 |  | -- | 45–64 | 2,564 | -- | -- | 65–74 | 6,122 | -- | -- |
| Boston [19] | 1988–1993 | 25–44 | 490 | 2.7 | -- | 45–64 | 1,004 | 2.6 | -- | -- | -- | -- | -- |
| Boston [21] | 2003–2008 | 25–44 | 586 | 2.2 | -- | 45–64 | 1,469 | 1.7 | -- | 65–84 | 2,353 | 2.6 | -- |
| New York [22] | 1987–1994 | 25–44 | 1,497 | 0.9 | 0.1-6.4 | 45–64 | 844 | 3 | 0.8, 11.1 | 65–74 | 4,895 | 1.3 | 0.3, 4.7 |
| New York [6] | 1990–2008 | 25–44 | 890 | 1.5 | 0.2-10.4 | 45–64 | 1,760 | 1.5 | 0.6, 3.5 | 65–74 | 3,555 | 1.7 | 0.6, 5.2 |
| Philadelphia [3] | 1985–1988 | 35–54 | 1,252 | 1.1 | 0.1-8.2 | -- | -- | -- | -- | 55–74 | 1,299 | 4.7 | 1.0, 22.3 |
| Toronto [20] | 1995–1997 | 18–44 | 515 | 2.6 | -- | 45–64 | 438 | 5.9 | -- | -- | -- | -- | -- |

One study [6] reported data for both homeless families and individuals who were homeless as single adults, with mortality rates for the latter group used in comparisons with members of the Housing First participant cohort

Abbreviations: CR, crude rate; CI, confidence interval; RR, rate ratio

^a^Deaths per 100,000 person-years of observation; obtained directly from previous studies or calculated using data published in each study

^b^Mortality rate ratios calculated by dividing the mortality rates for the HF participant cohort by corresponding mortality rates obtained from each published study; rate ratios are not race adjusted
